# Natural Food Colorants and Preservatives: A Review, a Demand, and a Challenge

**DOI:** 10.1021/acs.jafc.1c07533

**Published:** 2022-02-24

**Authors:** Cláudia Novais, Adriana K. Molina, Rui M. V. Abreu, Celestino Santo-Buelga, Isabel C. F. R. Ferreira, Carla Pereira, Lillian Barros

**Affiliations:** † Centro de Investigação de Montanha (CIMO), Instituto Politécnico de Bragança, Campus de Santa Apolónia, 5300-253 Bragança, Portugal; ‡ Grupo de Investigación en Polifenoles (GIP-USAL), Facultad de Farmacia, Campus Miguel de Unamuno s/n, Universidad de Salamanca, 37007 Salamanca, Spain

**Keywords:** natural food additives, colorants, preservatives, copigmentation, molecular dynamics

## Abstract

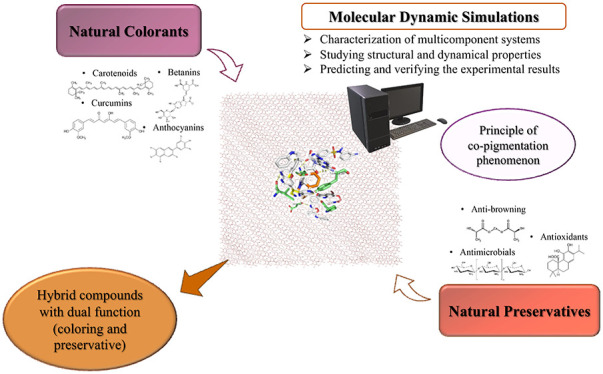

The looming urgency of feeding the growing world population along with the increasing consumers’ awareness and expectations have driven the evolution of food production systems and the processes and products applied in the food industry. Although substantial progress has been made on food additives, the controversy in which some of them are still shrouded has encouraged research on safer and healthier next generations. These additives can come from natural sources and confer numerous benefits for health, beyond serving the purpose of coloring or preserving, among others. As limiting factors, these additives are often related to stability, sustainability, and cost-effectiveness issues, which justify the need for innovative solutions. In this context, and with the advances witnessed in computers and computational methodologies for *in silico* experimental aid, the development of new safer and more efficient natural additives with dual functionality (colorant and preservative), for instance by the copigmentation phenomena, may be achieved more efficiently, circumventing the current difficulties.

## 1 Introduction

Improving food quality and safety is of paramount importance for human well-being. Since prehistoric times, man has been improving the diet and the way of hunting, domesticating animals and vegetables, preserving food by physical methods, and finally by adding molecules to food to enhance flavors or to preserve it.^1^

Over the years, several ingredients have performed useful functions in a diversity of foods, providing an affordable, nutritious, tasty, colorful, and safe food supply, with food additives and technology developments playing crucial roles.^2^ Their use in the food industry is fundamental, allowing loss reduction, quality increase, shelf life extension, new formulations development, and standardization, thus meeting the increasingly challenging market demands.^3^ Used in all types of foods, additives are becoming increasingly prevalent and important in human nutrition, being subjected to strict regulation, despite the controversy caused by conflicting results obtained in a large number of studies involving these compounds, along with different governments interpretations.^4^ Today, globally, hundreds of additives are added to food, while many others have been banned over the years.^1^

With an increasingly competitive market, it is essential to reduce production costs and monetize existing products, while ensuring food safety and quality. Since coloring and preserving additives are among the most important ones in the food industry, improving the appearance and preservation of foodstuffs, several studies have been focusing on finding new solutions and/or improving existing alternatives. Finding compounds that can have both capacities (colorant and preservative) and, additionally, exert bioactive functions can be a promising solution. However, to obtain benefits such as antioxidant or antimicrobial activity, the concentration of the compounds commonly used as dyes are usually high, not meeting the requirement of the admissible daily intake (ADI).^5^ Research and development of new molecules through new chemical approaches, with the modification of natural molecules already known, so that they can develop a better and double performance (colorant plus preservative), may be a path to be followed by the scientific community to circumvent the difficulties and monetize the use of these additive molecules in the food industry.

### 1.1 Food Additives, Food Industries, and Their Demands

The use of pesticides, irradiation, food additives, and even trace substances in the production and preservation of food, has generated a high concern among consumers, due to the possible short, medium, and long-term health effects.^6^

These issues and health concerns are even more obvious in highly processed and modified food products, which have been rising in the last decades and hold a higher additive load. Currently, there is a general pressure for innovation in the food and biotechnology industries in order to provide food products adapted to the modern lifestyle (highly attractive and perfect foods, with particular organoleptic characteristics as well as a long shelf life) but, at the same time, healthy and safe food.^7−9^

To meet most of these conditions, various processes and ingredients such as food additives (colors, sweeteners, preservatives, emulsifiers, acidifiers, etc.) have been used, which have been developed both to prevent food spoilage and to improve its taste and appearance. However, studies of their long-term effects are still needed.^10^

As a principle, food additives should only be employed if their technological effect is justifiable and cannot be met by other feasible methods. Moreover, these compounds must not pose a health risk to consumers at the level of use proposed.^11^ The most important regulatory bodies that set the conditions, legislate, and oversee the approval and standardization of food additives in the world are EFSA, the European Food Safety Authority, in the European Union (EU), and the FDA, Food and Drug Administration, in the United States (U.S.). Also, JECFA, the Joint Expert Committee on Food Additives of the Food and Agriculture Organization (FAO)/World Health Organization (WHO), and the Codex Alimentarius are also key bodies responsible for carrying out studies and safety risk assessments and issuing declarations. Codex Alimentarius is the point of reference for other official food authorities around the world for meeting national and international quality standards in the export of processed foods.^11^ In the EU, the classification of food additives depends on the function in food, which is divided into 26 functional classes.^10^ On the other hand, in the United States, additives are reduced into 2 or more classes, where 3000 are allowed by the FDA, which are classified into preservatives, nutritional additives, colorants, flavorings, texturizers, and miscellaneous agents.^13^

In Europe, all food additives are given a specific code that begins with the letter E followed by three or four digits, referred to as the E-number, making it easier for the consumer to understand the label of foodstuffs from different European countries. The added amount of additive is scrupulously calculated for the type of food, so as not to exceed the acceptable daily intake (ADI), which is the cumulative amount of a specific additive that, consumed daily, has no harmful effect on health. The same code number is also used by the Codex Alimentarius.^14^

However, despite their manifest benefits, it is worth noting that a large proportion of approved food additives are synthetic in nature, and when used incorrectly, a range of side effects, toxicity, and other adverse reactions have carcinogenic capacities.^9^ In this regard, regulatory agencies worldwide have carried out extremely stringent evaluation procedures, from which several synthetic additives have been banished due to gastrointestinal, respiratory, dermatological, and neurological adverse reactions.^15−18^

However, a global consensus on food additive legislation has not yet been established. Some substances can be added to foods in the U.S. and banned in the EU, such as the antimicrobials sodium sorbate (E201) and calcium sorbate (E203) and the colors FD&C green no. 3 (Fast Green (E143)) and citric red no. 2 (E121). On the other hand, the antimicrobial sodium methyl *p*-hydroxybenzoate (E219) and the coloring agents carmoisine (E122), amaranth (E123), and patent blue (E131) are allowed in the EU and banned in the U.S.^11,13^ With all this, there are contradictions in the legislation, which leads to problems in food safety and obstacles to international trade. Likewise, there is no established definition for preservatives, antioxidants, colorants, or natural sweeteners; only natural flavorings have the same legislation in both the EU and the U.S., which has led to erroneous use at a general level for all classes of additives, thus giving erroneous interpretations of what is natural and what is synthetic. For all these reasons, the need for a uniform legislation on natural additives is evident, due to their wide interest, especially in developed countries.^19^

At the same time, there is a growing demand for food products with health promotion and disease prevention capacity.^6,20,21^ They are known as functional foods, with a currently accepted worldwide conceptual definition as foods that “have been satisfactorily shown to affect one or more target functions in the body, in addition to appropriate nutritional effects, in a way that is relevant to an improved state of health and well-being and/or reduced risk of disease”.^22^ In this sense, natural additives may also be included in this concept as they possess a high biological quality and are able to present health-promoting abilities.

## 2 Food Colorants

Organoleptic characteristics largely determine the acceptance, selection, and subsequent consumption of foods. Color can be considered one of the most impressive and charming attributes of foods, and although natural food products have their own color, the different processes they undergo and factors, such as the presence or absence of oxygen, metals, light, pH, and water activity, can produce undesirable modifications. To circumvent this problem, chemical compounds that impact color are intensely used by food industries, while an increasingly strict and regulatory legislation accompanies these advances to ensure good manufacturing practices and total consumer safety.^23−9^

A color additive, or food colorant, is according to the FDA, “any colorant, pigment or substance that, when added or applied to a food, drug or cosmetic, or to the human body, is capable (alone or through reactions with other substances) of imparting color”.^26^

Differences in chemical structures, sources, and purpose of use can make classifications of colorants complex, and they can be classified according to several criteria: origin (natural, natural-identical, or synthetic; organic and inorganic), solubility (soluble and insoluble), and hiding power (transparent and opaque). The most used form is according to their origin, either natural or artificial. Natural dyes can be obtained from plant tissue (e.g., curcumin, carotenoids, anthocyanins, betalains, or chlorophylls), animal cells (carminic acid and kermesic acid), metabolism of microorganisms (carotenoids and chlorophylls), or mineral sources (titanium dioxide or calcium carbonate). Artificial colorants are achieved by chemical synthesis, and they are not found in nature.^27^

All these dyes do not impart an aftertaste when added at a certain concentration, and their use in the food industry is mainly in confectionery, bakery, beverages, dairy products, and meat.^28^

### 2.1 Synthetic Colorants

Preferred by the industry, synthetic colorants are chemically or physically modified products with desirable characteristics for manufacturing, such as high purity, coloring capacity, stability, brightness, wide range of shades, uniformity and reproducibility in production, and low cost compared to natural colorants.^14,9^

As science has advanced, more artificial colors have been developed that are very stable and allow a wide range of colors, which has led to their increasing use in the food industry. However, despite the continuous research for synthetic compounds, many of them are for humans, and based on the latest discoveries regarding the side effects and toxicity problems of some synthetic dyes, natural alternatives have become more appealing for consumers worldwide.^5^ Furthermore, in order to detect the presence of synthetic food colors in food, highly specific methods and procedures have been developed, which also make it possible to determine the probability of side effects and toxicity in the medium- and long-terms.^29^

Studies have reported that the intake of artificial colorings, mainly nitro derivatives, of the azo type (E102, E110, E122, E123, E124, and E129) can cause some health problems. The European Parliament, in 2008, decreed those foods containing one or more of these color additives must be labeled with the name or the E-number information followed by the warning “may have an adverse effect on activity and attention in children”.^13^ Tartrazine (E102), a lemon yellow, used in candies, ice cream, cereals, soups, jellies, cakes, soft drinks, and other foods, is one of the most controversial color additives regarding its safety. Obsessive-compulsive disorders and hyperactivity in children have been linked to its intake, as well as its mutagenic capacity, due to its interaction with human serum proteins.^17,18,30^ In recent studies, administration of tartrazine at ADI levels in mice showed increased lipid oxidation and changes in biochemical markers in brain tissue hematotoxin,^31^ immunotoxin effects, renal disorder, and increased DNA abnormalities.^30,32^ Sunset yellow (E110), obtained from petroleum-derived aromatic compounds, has been related to increased pro-inflammatory activity, as have carmoisine (E122), allura red (E129), and ponceau 4R (E124), which also bind to human and bovine serum albumin.^18^ Amaranth (E123), a dye that imparts red color to foods such as in candies, ice cream, and beverages, allowed in the EU but banned in the U.S. due to carcinogenicity, has shown a high genotoxic effect on cultured human lymphocyte.^33^ It is important to note that there are colors that are permitted in the European Union, but the same is no longer the case in the United States.^34^ Given these contradictions and the impact of artificial colorings in health, more and more emphasis is being placed on research on sources of natural origin, both for their safety and health-promoting properties.^35−37^

Despite the controversy surrounding food colors, there is high pressure on the search for natural colors because of the role they play in the food industry. In the U.S. and the EU, some alternatives have been developed with excellent results, among which molecules such as carotenoids, anthocyanins, annatto, and paprika stand out with possible characteristics that could substitute their synthetic counterparts. However, these compounds have disadvantages such as instability with respect to pH and temperature, loss of color due to oxidation, higher manufacturing cost, and greater quantity of the product with respect to synthetic colorants.^38^

### 2.2 Natural Colorants

Until the middle of the last century, the dyes used in food were of natural origin, for example, saffron (obtained from stigma and styles of the flower of *Crocus sativa* L.), orcein (extracted from certain lichens), cochineal (obtained from certain insects of the family *Coccidae*, a parasite of some cacti), caramel (sugar paste turned into syrup), red beet (aqueous extract of the red beet root), alizarin (obtained from tropical woods), and indigo (from the indigo plant or glasto, a European shrub).^14,28^ Today methods of collection, extraction, purification, stabilization, and standardization for various natural food colors are available, such as anthocyanins, betalains, chlorophylls, carotenoids, etc. These include different groups of chemical compounds that can be used directly as dyes or in chemically modified forms to produce different hues, ranging from green to yellow, orange, red, blue, and violet, depending on the compounds or their stabilized forms.^9,39^

**Figure 1:**
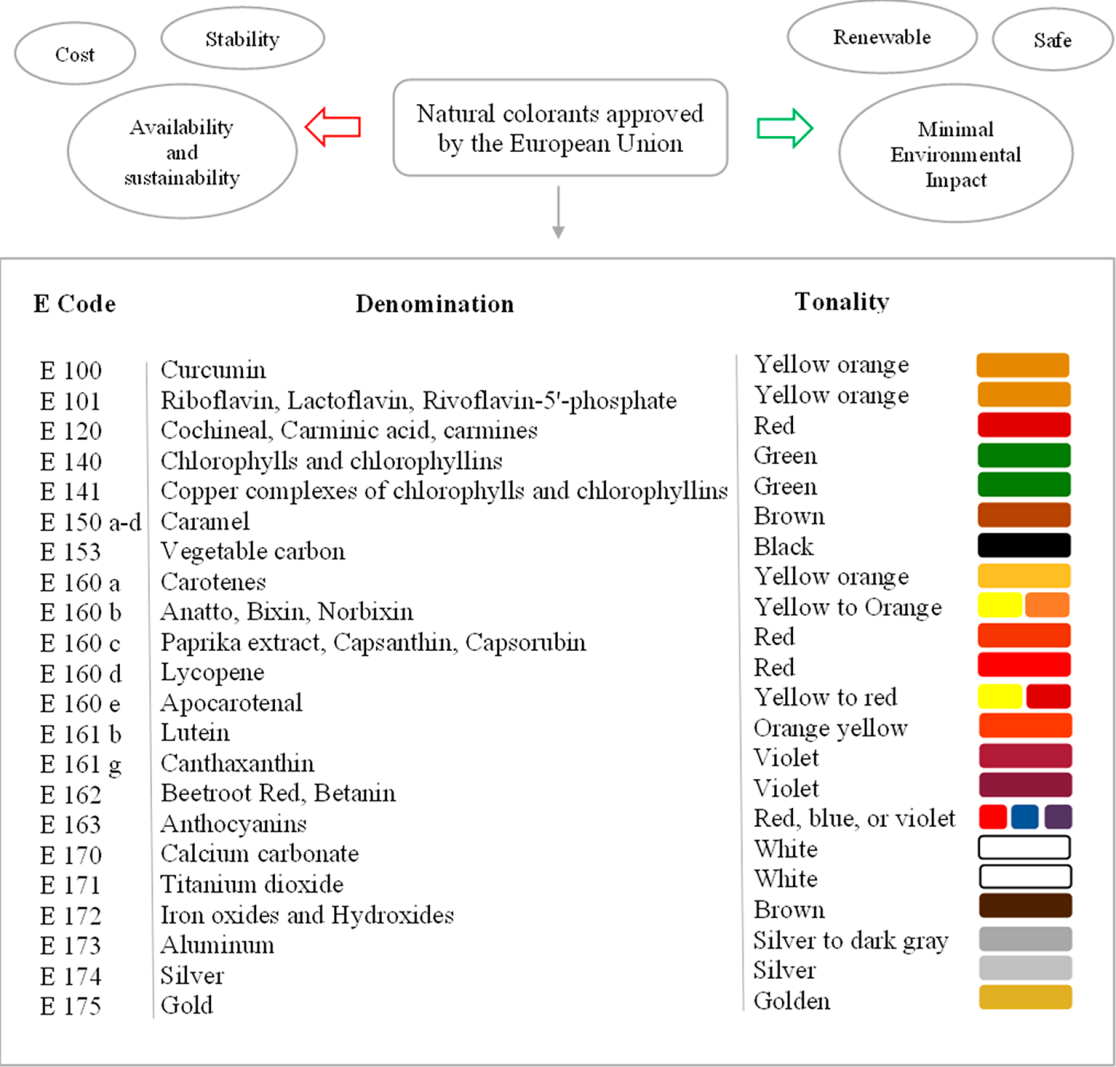
Natural colorants approved by the European Union, their shade, and functionality.

Curcumin (E100), a purified turmeric pigment extracted from the dried rhizomes of *Curcuma longa* L., is a widely used food coloring that imparts orange color and is used in mustard, yogurt, bakery products, dairy products, ice cream, and salad dressings.^40^ Carminic acid (E120) is the main pigment present in the insect *Dactylopius coccus* Costa (cochineal), which when complexed with aluminum, turns into bright red. This dye is quite expensive compared to other natural reds, such as anthocyanins, although it is considered technologically important due to its stability. It replaces the use of potentially toxic synthetic dyes; however, the food product cannot be classified as vegan/vegetarian or kosher because it contains an animal product.^41^ It is used in jams, jellies, bakery products, dairy products, and noncarbonated beverages.^40^ Chlorophylls and chlorophyllins (E140) occur naturally in plants; of the different existing chlorophylls, only two are used in the food industry as colorants, mainly due to their difficult stabilization; more stable copper complexes of them are also authorized (E141). The commercial chlorophyll dyes used are extracted from alfalfa and have been employed in dairy products, soups, beverages, and sugar confectionery.^40^ Annatto (E160b), extracted from the seeds of the fruits of *Bixa orellana* L. tree, has the carotenoids bixin and norbixin as its main constituents, which are yellow to orange and provides a slightly redder color than β-carotene. It is used in cakes, cookies, rice, dairy products, flour, fish, soft drinks, snacks, and meat products.^41−43^ Paprika extract, capsanthin, and capsorubin (E160c) also consist of carotenoids, with an orange to red color. Many other carotenoids used in food, namely, β-carotene, lutein, violaxanthin, neoxanthin, β-cryptoxanthin, fucoxanthin, lycopene, and astaxanthin, are mainly applied to sauces, marinades, seasoning mixtures, toppings, beverages, milk, and others.^19^ The main anthocyanins (E163) in nature are glycosides of the aglycones cyanidin, delphinidin, malvidin, pelargonidin, peonidin, petunidin, with colors ranging from red, purple, violet, and blue, with over 700 anthocyanins having been identified.^44−46^ Their main uses are in soft drinks, confectionery, and fruit preparations.^19^ Betalains are pigments with similar colors to anthocyanins, ranging from red-violet (betacyanins) to yellow-orange (betaxanthins). However, betanin (E162) derived from beets is the only betalain with legislated use and has been the most massively utilized natural pigment in industry to replace Allura red AC (E129; FD&C Red 40).^41^ Its use is approved to provide a red hue to candies, ice cream, meat substitutes, and beverages.^34,47^ Despite the limitations of using betalains as a food dye due to their sensitivity to light and heat, they have several advantages over other natural dyes, such as higher water solubility and strong dyeability and stability in neutral and acidic pHs, and given their range of colors, there is a great potential to create a gradient of natural color shades from these pigments.^9,41,48,49^ All natural dyes approved by the European Union are collected in Figure 1.

An alternative to natural blue dyes would be the phycocyanin pigments from Spirulina (*A. platensis*). The search for a vibrant and stable source of natural blue dyes has been stealthy, and since anthocyanins, which may have a blue color between pH 5–7, tend to change their hue depending on the pH value and water content, these would be a good alternative.^50^ Spirulina, a Gram-negative cyanobacterium, produces picocyanobacteria that can be isolated, and phycocyanin can be recovered as a phycocyanin protein complex. Currently, picocyanobacteria from Spirulina provide the only natural blue dye approved in the United States and Asia, although it is not yet allowed in the European Union.^51,52^ Initially, phycocyanins were used only to color sweets and chewing gums, but innovations in food processing have allowed the range of phycocyanin-colored products to be extended to dairy products, soft drinks, and cosmetics.^53^ They have been mainly applied in beverage products, but low pH stability issues continue to limit a wider application.

Another approach to produce natural dyes would be microorganisms such as bacteria and fungi. These can generate a range of natural pigments that botanical sources are unable to provide, they are less constrained by seasonal variation issues, and their extraction can be simplified and easily scaled to industries.^54,55^ Bacteria, typically easier to modify genetically, with a shorter life cycle, are an opportunity to expand research in pigment production, which is still poorly understood.^56^ A laboratory at Rensselaer Polytechnic Institute in Troy, New York, has developed a way to use *E. coli* and glucose to produce anthocyanins at industrially relevant levels of g/L.^57^ Regarding fungi, nontoxic *Monascus sp*., which produces six large polyethylene pigments ranging from yellow to red, has so far not gained U.S. or EU approval due to concerns over citrinin production (a potent fungal toxin), although it has been used successfully in Asia for hundreds of years.^54,56^ However, there are concerns with customer acceptance of food coloring coming from microbes.

Despite the instability associated with these types of dyes, some natural food colors have proven to be as effective as those derived from chemical synthesis, with the subsequent benefits of being safer and providing health benefits, in addition to conferring organoleptic characteristics, thus exerting two or more benefits as food ingredients. In fact, several food additives exerting coloring effects also act as antioxidants and even preservatives, also conferring functional properties to food products.^58,59^ The consumption of functional foods has been increasing, due to the healthy properties that they provide to the consumer when they are adequately included in the daily diet.^22,59,60^

Many companies have been substituting artificial colorants for natural ones in order to satisfy consumers’ demand, due to the fact that they not only have the capacity of pigmentation but also have health properties.^28,38,59^

It should be emphasized that, in general, the stability can be understood as one of the most important factors for the use of natural food colorants, since unpleasant organoleptic characteristics appear when unstable food colors are applied, leading to food rejection by consumers.^5^ Thus, several approaches have been developed to solve this problem,^7,47,61^ while other studies have focused on finding different sources of promising food colorants, so that a growing and emerging number of studies are seeking to explore new sources of natural colorants, actually there are already several available reports exploring the potential of novel alternatives from plants, insects, fungi, bacteria, and algae.^62−66^ For that purpose, extraction and stabilization techniques have also been assessed to allow their application in the food industry, in order to compete with artificial solutions in terms of coloring efficiency and color stability over the products storage time.

## 3 Food Preservatives

Food products are susceptible to deterioration by microbiological, enzymatic, physical, or chemical processes, which can reduce their quality, nutritional value, and safety as well as lead to undesirable changes in their physicochemical and sensorial attributes. With this in mind, a variety of biological, physical, and chemical methods for food preservation have been developed to extend the shelf life of foods and keep them safe for consumers, without modifying their sensory characteristics.^67^ The application of preservatives in food processing is one of the current food preservation techniques which, besides maintaining the quality of food, helps control contaminations that can lead to foodborne diseases. They can be natural or synthetic substances and be subdivided into three groups: antimicrobials, antioxidants, and antibrowning agents.^68^ Antimicrobials have been applied to control and prevent natural spoilage by microorganisms; antioxidants are used as preservatives to limit or delay biological and chemical spoilage of foods by preventing auto-oxidation of pigments, flavors, lipids, and vitamins; and last, antibrowning agents are used to prevent browning of foods, which can occur at any time during handling, processing, and storage.^69^

### 3.1 Antimicrobials

Antimicrobial compounds can be naturally present in foods or added to delay and/or prevent the proliferation and growth of natural microorganisms responsible for the spoilage of foods (bacteria, yeasts, and molds) as well as to prevent/control contamination by pathogenic microorganisms, thus ensuring food safety and quality.^70^

The use of antimicrobial additives increases the shelf life of food, but in high concentrations, these compounds can promote an unpleasant taste, strong odor, altered viscosity, and color retention, with their choice dependent on the antimicrobial properties and spectrum of activity, the physicochemical composition and properties of the food matrix, and the nature of the storage and preservation methods.^38,71^ They can be either natural or synthetic.

#### 3.1.1 Synthetic Antimicrobials

Several artificial preservatives have been officially recognized by the regulatory community to be used as food antimicrobials.^72^ These are substances of chemical origin that prevent or inhibit the proliferation and growth of bacteria, yeasts, and molds. Among those approved for food applications, inorganic acids and their sodium salts, such as nitrite and sulfate, and weak organic acids, such as benzoic acid, sodium benzoate, sodium propionate, and others, are included.^73^ Despite the beneficial effects and important role of these compounds in the preservation of food products, the use of chemical antimicrobials in food is still a subject of discussion and acknowledged controversy, as the results of reported research do not demonstrate consistency. Indeed, it is not simple to provide clear conclusions on efficacy and safety, and synthetic antimicrobial food additives, such as sorbates, nitrates, and sulfites, have demonstrated various side impacts on human health.^74^ Although the reports are disturbing, these are indispensable in the food industry, and the only way to reduce their application would be to find a nontoxic substitute with the same preserving effect. The use of these compounds decreases the incidence of food spoilage and food poisoning.^75^ Long-term exposure to sodium benzoate (E211) is claimed to be nonhazardous, but this has yet to be proven, and while some researchers consider sodium sulfate (E221) to be hazardous to health, others say otherwise. Sodium sorbate continues to raise questions and concerns; some studies have shown that it may be genotoxic to human lymphocytes *in vitro*, depending on the dose used.^76,77^ Over the years, extensive studies have been conducted on sorbates (E200–E203) and their health implications, in which they have been described as possible genotoxic and mutagenic, but other studies report that this is not relevant.^78−80^ Also, parabens (E214–E218), generic name of the group of antimicrobials that are alkyl esters of *p*-hydroxybenzoic acid, have been widely used due to the absence of odor and/or taste; however, it has been shown that the use of parabens can induce migratory and invasive activity in human breast cancer cells *in vitro*.^81,82^ Nitrites (E249 and E250) and nitrates (E251 and E252) are other antimicrobials used in food. The second group has recently been restricted in the EU and can only be added to meat for slow curing. Nitrites are used in meat for color formation, flavor enhancement, and antimicrobial activity and are the only food additives able to inhibit botulinum toxin, justifying their use in the food industry by their benefit/risk, despite having shown carcinogenic effects, among other harmful effects to humans.^83^ In the European Union, their use is only permitted in the lowest possible dosage. Nitrates and nitrites can also be found naturally in untreated fruits and vegetables and may also be involved in the formation of nitrosamines.^84−90^ Sulfites (E220–228) are used in foods such as wine, dried fruit, dehydrated cookies, and fish, among others and can act as such or combined with organic acids. However, they are known to cause adverse reactions in sensitive people and have shown cytotoxic and carcinogenic effects for rats and humans; also they are known to induce deterioration of thiamine (vitamin B1) in foods.^91−93^ Propionic acid (E280) and its sodium (E281), calcium (E282), and potassium salts (E283) are used primarily in bakery products to prevent mold and other fungal contamination. There are not many studies on their toxicity, although they have been reported to induce onion root tip abnormalities, producing chromotoxic effects and, consequently, chromosomal anomalies and have also been linked to irritability, restlessness, inattention, and sleep disturbances in some children.^13,74^

Other effective antimicrobial agents are acetic, benzoic, and lactic acids. Malic (E296), fumaric (E297), citric (E330), and other organic acids are also used, having, however, limited antimicrobial activity; in fact, they are more often used as flavoring agents. Generally, the mechanisms of action of weak organic acids are based on the inhibition of nutrients.^74^ Acetic acid (E260), the main component of vinegar, is commonly used in condiments including mustard, salad dressings, and mayonnaise to inhibit the growth of fungi and bacteria. It is more active against bacteria and yeasts than in molds. Benzoic acid and sodium benzoate (E210 and E211) tend to be used in foods where the pH is low, such as fruits, mayonnaises, pickled vegetables, and beverages, exerting their antimicrobial activity against yeasts and fungi, including inhibition of aflatoxin production. Lactic acid (E270) is incorporated into acidified food products to provide additional stability and safety. It is effective in reducing microbial communities on meat surfaces and is even more efficient than acetic acid in reducing *E. coli* O157:H7.^74^

#### 3.1.2 Natural Antimicrobials

**Figure 2:**
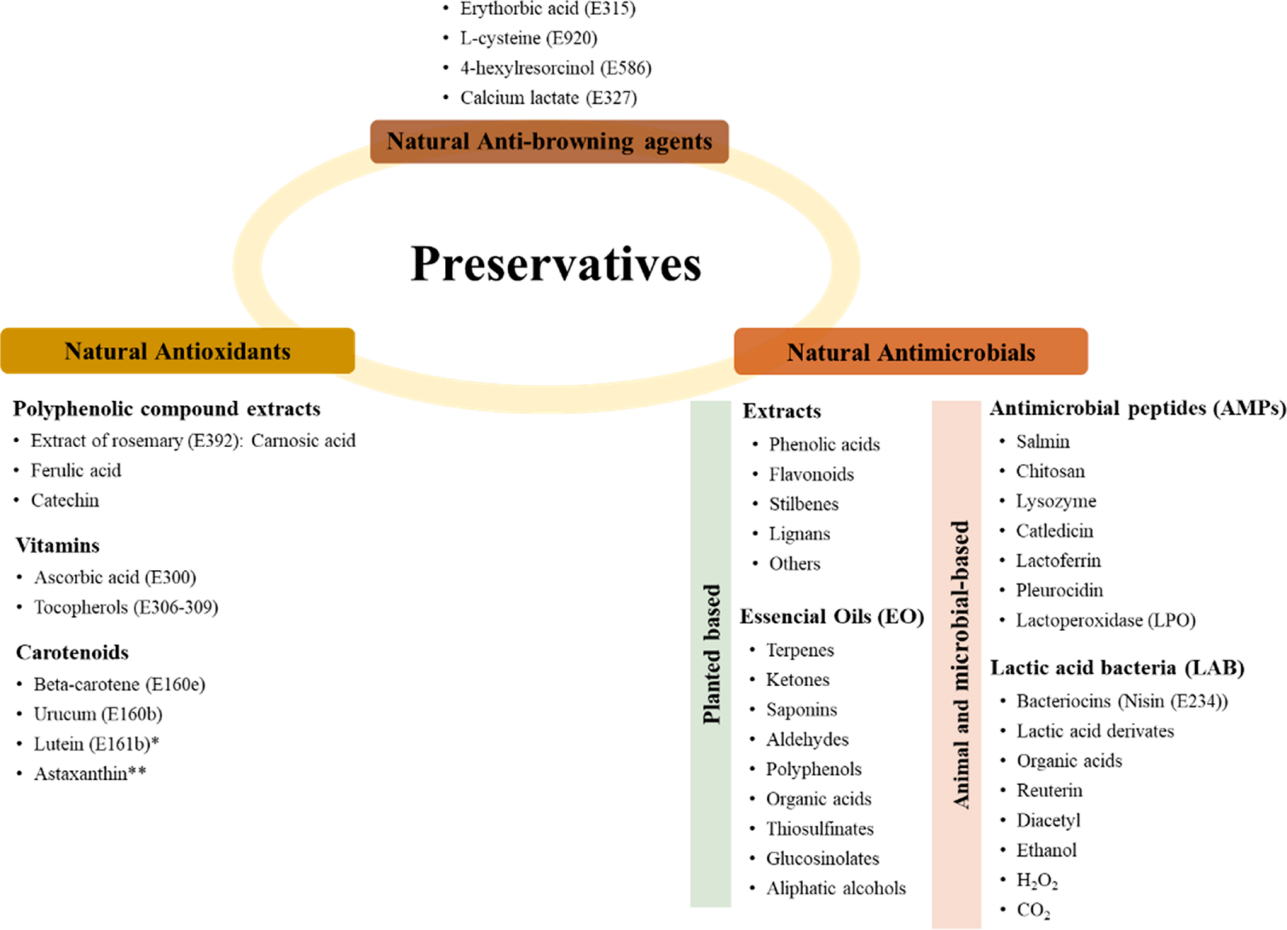
Natural compounds with preservative capacity, in use or under research. *Additive allowed as a colorant but not as an antioxidant. **Additive still under study, not yet permitted in the EU.

Due to the potential of synthetic preservatives to cause health problems, consumers and companies are trying to replace synthetic preservatives with natural preservatives (Figure 2), which can be achieved from sources such as plants, bacteria, fungi, animals, and algae and are considered safer for humans and the environment.^68^

##### 3.1.2.1 Plant-Based Antimicrobials

Depending on the type, nature, and concentration, various plant extracts (e.g., herbs and spices) have preservative aptitudes, with antimicrobial activity against various microorganisms and can thus increase the storage life of foods.^68^ Thanks to compounds, such as phenols, alcohols, aldehydes, ketones, among others, these can be alternative sources of new antimicrobial compounds, having already been reported by several researchers.^94−97^ Antimicrobial compounds present in plants include essential oils, phenolic compounds, polypeptides, lectins, and alkaloids. Additional categories of substances, such as polyamines, organic acids, glycosides, and glucosinolates, have also shown potential as natural antimicrobials.^74^

Essential oils (EO) are produced mainly by aromatic plants and are bioactive, complex liquid volatile compounds characterized by an intense flavor and smell. In the food industry, they are mainly used as flavoring agents; however, they may also act as natural antioxidants and antimicrobials in food preservation. The principal plants used for EO extraction include lemongrass, peppermint, lavender, geranium, thyme, oregano, sage, rosemary, basil, vanilla, clove, fennel, cumin, cinnamon, and anise. The main compounds present in EO that confer antimicrobial activity are terpenes, aldehydes, ketones, aliphatic alcohols, organic acids, phenolic compounds, saponins, glucosinolates, and thiosulfinates.^100^ Rich in terpenes, such as linalool, eugenol (from clove and cinnamon), thymol (from thyme and oregano), carvone, carvacrol (from oregano), citral, limonene and their precursors, and many other substances, EO possesses antimicrobial properties against a broad spectrum of food-borne microorganisms, including spoilage and pathogenic bacteria and fungi.^94,101^ Carvacrol, for example, found in high concentrations in oregano, has antimicrobial and antifungal power, even at low concentrations, and these effects can be enhanced if cinnamaldehyde and nisin are also present. Thymol, an isomer of carvacrol, shows the same antibacterial and fungicidal activity, including inhibitory effects against *Aspergillus* species *in vitro*.^102^ Ozogul et al.^103^ revealed that an EO-based nanoemulsion (thyme and rosemary) can be used as a preservative ingredient due to its capacity to improve microbiological and organoleptic properties in rainbow trout fillets. Generally, EO produces a better inhibitory effect against Gram-positive than Gram-negative bacteria, since they are lipophilic compounds and can penetrate Gram-positive bacteria.^104^ Their capabilities can be further enhanced by synergism with bacteriocins or even food constituents, depending on the purpose. While essential oils are generally incorporated into foods by direct mixing, immersion, and spraying methods, they recently found application in bioactive packaging, encapsulation, and nanotechnology. Other considered applications are edible films and coatings with incorporated bioactive substances, which have been targeted especially in food preservation, acting as carriers for EO and thus controlling microbial growth.^68^ In this context, the EO of *Origanum majorana* encapsulated in terpinen-4-ol enriched chitosan nanoemulsion has been shown to be a considerable preservative of stored food products against contamination by fungi, aflatoxin B1 (AFB1), and lipid peroxidation.^105^ Consequently, these bioactive encapsulated compounds could be approved as a suitable antifungal agent to extend the shelf life of food products. Furthermore, the use of EO of clove, oregano, sage, ginger, and thyme, alone or in combination, has demonstrated the ability to limit the oxidative process in food matrixes, such as meats and dairy products, due to their richness in phenolic compounds. In this regard, various EO have shown remarkable antioxidant activity, being considered effective in retarding lipid oxidation in fish and meat products and applied as preservatives in fish supplements.^106^ The only drawback of EOs is that, even at low concentrations, they can be toxic to humans, so more extensive studies should be conducted to determine their actual effects on the human body and to establish an ADI.^107^ On the other hand, weak organic acids (citric, acetic, malic, lactic, tartaric, etc.), present in oranges, lemons, apples, and grapes, along with other fruits and juices, are natural flavoring and acidifying agents in acidified foods and can prevent various contaminating microbes.^74^

##### 3.1.2.2 Animal and Microbial-Based Antimicrobials

Other bioactive compounds that can be applied in food preservation are those derived from animal secretions or defense systems. Antimicrobial peptides (AMPs), for example, are oligopeptides with a broad spectrum of activity against bacteria, fungi, protozoa, and some viruses. They are isolated from natural sources such as plants, insects, amphibians, crustaceans, and marine organisms, and those already used as food additives have been claimed to possess positive health effects with unreported toxicity.^68^ Lysozyme, lactoferrin, ovotransferrin, pleurocidin, defensins, chitosan, etc. are other possible antimicrobials of animal origin. Lysozyme, from various sources, has antimicrobial activity, particularly against Gram-positive bacteria. This enzyme is broadly used as preservative in meat, fish, milk, and dairy products as well as fruits and vegetables, although it has a limited action against bacteria and fungi. Its activity can be increased when used in synergy with other preservatives, as nisin.^13^ Lactoferrin has iron-binding capacity and is found in milk and other secretions. Two AMPs isolated from fish are pleurocidin and protamine, which have activity against the bacterium *Listeria monocytogenes* as well as other food-borne microorganisms. Cathledicin, found in mammals, shows activity against bacteria, fungi, and viruses.^108^ Salmin, a cationic AMP derived from salmon milt, was found to slow the growth of *Listeria monocytogenes* in smoked salmon.^109^ The use of chitosan and its derivatives (extracted from the exoskeletons of crustaceans and arthropods) in the food and pharmaceutical industries is mainly due to their preservative effects. They can find application in the development of edible films and coatings used in the food industry and are also employed in combination with other molecules such as xylan and glucose.^110^ In fact, chitosan fibers combined with flavonoids have shown excellent antioxidant activity.^111^ The application of chitosan in meat and seafood conferred significant oxidative stability by reducing the thiobarbituric acid value and retarding lipid oxidation during storage.^67^ One enzyme that has attracted attention for its bactericidal or bacteriostatic properties is lactoperoxidase (LPO), due to its use as a natural antimicrobial agent in food packaging. The application of chitosan with LPO to coat trout fillets preserved their quality.^112^ Likewise, incorporating LPO in alginate coatings had an inhibitory effect on *Listeria monocytogenes* and *E. coli* bacteria in rainbow trout fillets.^113^

Beyond the demonstrated activity of AMPs against various food-borne pathogens,^114^ many research studies have also reported that protein hydrolysates and peptides obtained from goat milk, blue mussel, Pacific hake fillet, tuna backbone, or beef can exert a higher antioxidant activity than α-tocopherol, BHT and BHA (butylated hydroxytoluene and butylated hydroxyanisole, respectively) additives, being therefore valuable alternative preservative and functional ingredients to be used in food to decrease oxidative change during storage.^111,115^

Food biopreservation using microbes and microbial metabolites to increase food safety and extend shelf life is one of the alternatives to chemical treatments. Lactic acid bacteria (LAB) are the main candidates, and several species have been used as cultures to produce fermented products such as sausages, cheeses, or yogurts. Antibacterial compounds such as bacteriocins, organic acids, reuterin, diacetyl, ethanol, CO_2_, H_2_O_2_, and lactic acid derivatives are generated by LAB.^74^

Bacteriocins are ribosomally synthesized antimicrobial peptides that are produced by bacteria and have been employed in the preservation of meat and vegetable products for having bactericidal or bacteriostatic effects.^100,116^ Bacteriocins used in food are synthesized by strains of *Carnobacterium*, *Lactococcus*, *Lactobacillus*, *Pediococcus*, *Leuconostoc*, and *Propionibacterium* and the major bacteriocins include nisin, pediocin, diplococcin, plantaricin, acetylphilin, helveticin, bulgarican, and lactacin.^74^ They can also be used in bioactive packaging applications to control food pathogens by preventing the growth of microorganisms on the food surface through contact of the packaging material.^117^ Nisin is an antimicrobial peptide naturally synthesized by *Lactococcus lactis* and is the only bacteriocin recommended for global application as an additive in the food industry with GRAS (generally recognized as safe) status and excellent antibacterial properties.^118^ It has activity against Gram-positive bacteria, including *S. aureus* and *L. monocytogenes* and limits the growth of spores of several *Clostridium* and *Bacillus* species. It has little effect on Gram-negative bacteria, yeasts, and molds,^119^ but the incorporation of chelating agents (e.g., EDTA) may enhance its effect. It has been used with good results in meat, cheese, and dairy products, seafood, and in the wine and beer industries, being able to tolerate high and low temperatures and acidity. Furthermore, it has also demonstrated ability to prevent the progression of human squamous carcinoma cell lines, although it also showed toxicological effects in mice.^68,120^ The synergistic effects of nisin with carvacrol and lysozyme, as well as its encapsulation feasibility, have been explored with visible success.^13,102,121−123^

On the other hand, reuterin appears as a broad-spectrum antimicrobial substance, produced exclusively by the enzyme glycerol dehydratase from *L. reuteri*. The high-water solubility, heat resistance, stability over a wide range of pH values, and lipolytic and proteolytic enzymes make it a good biopreservative for foods. It controls Gram-positive and Gram-negative spoilage and pathogenic bacteria in milk, dairy, and meat products and can prevent the growth of *Aeromonas hydrophila*, *L. monocytogenes*, *E. coli O157: H7*, *Clostridium jejuni*, *Y. enterocolitica*, and *S. aureus*.^124^

Organic acids play an important role in the ability of LAB starter cultures to inhibit undesirable microorganisms, providing food safety. They cause a pH reduction, inhibiting spoilage and pathogenic bacteria.^125^ Lactic acid is the main compound produced in food fermentations, followed by acetic and propionic acids. *Enterococcus*, *Lactococcus*, *Lactobacillus*, *Streptococcus*, *Pediococcus*, and *Leuconostoc* are some of the strains that provide lactic acid as the main product, and yogurts, olives, sauerkrauts, and fermented sausages are mainly maintained by this acid.^126^ Propionic acid is generated by *Propionibacterium* in cheese.^74^ The hydrogen peroxide produced by LAB is effective against spoilage and pathogenic microorganisms such as *Pseudomonas spp*. and *S. aureus*. Carbon dioxide is also generated during fermentation of vegetables, such as sauerkraut, and helps to establish anaerobic conditions, being lethal to certain aerobic food microorganisms and having the ability to lower intracellular pH and inhibit enzymatic reactions. It generally prevents the growth of obligate aerobes, such as fungi, and slows the growth of facultative microorganisms, such as *Enterobacteriaceae*.^127^ Diacetyl generated by strains from many LAB has antibacterial activity against yeasts, fungi, and Gram-negative bacteria, which are more susceptible to diacetyl than Gram-positive bacteria.^128^ Despite being negligible to biopreservation, the contribution of ethanol can increase the lethal effect of low pH and lactic acid in *E. coli*, although at higher concentrations than those likely to occur in lactic fermentations.^129^

As described, there is a wide variety of natural antimicrobial compounds. However, some have not yet been approved as food antimicrobials, in part due to the extensive toxicological experiments required to ensure their safety. Consequently, additional research is needed to discover the optimal levels of antioxidants and antimicrobial substances that can be used harmlessly in food systems without excessively altering sensory or physicochemical characteristic.^130^

### 3.2 Antioxidants

In the storage process, two types of oxidations (lipid peroxidation and rancidification) occur in foods. Molecular oxygen oxidation is the primary degradation process in foods; it promotes a loss of nutritional value and produces an undesirable taste and smell.^23^ Auto-oxidation in foods can occur in vitamins, but it is mostly observed in fat (unsaturated fatty acids, cholesterol, and phospholipids).^131^ Beyond the impacts in the organoleptic properties, such as the alteration of the color and texture and the induction of a rancid taste, it also leads to the formation of toxic compounds.^19,132^ To prevent or delay these reactions, the food industry relies on antioxidants, which scavenge free radicals and oxygen, stopping peroxidation at the initiation or propagation stages, thus prolonging the shelf life of foods and preventing their decomposition without adding taste or odor to the food or changing its appearance.^133^

The importance of separating synthetic/artificial food additives from natural food additives is becoming more and more necessary due to the interest in the application of the latter. Likewise, the amounts and permissions of use of natural and synthetic antioxidant sources in each type of food should be added to the official tables.^134^

The main synthetic antioxidants used, mainly to inhibit the oxidation of fatty acids, are BHA (E320) and BHT (E221). BHA is used for foods and coatings, working better in animal fats than in vegetable fats. The acceptable daily intake (ADI) is 1 mg/kg body weight (bw) and exposure of children and adults should not exceed these doses. For its part, BHT, one of the cheapest antioxidants to produce, is generally used in animal fats and dry breakfast cereals, not exceeding an ADI of 0.5 mg/kg bw.^135^ These antioxidants have synergistic behavior when added to foods, deeply volatile given their chemical structures, and methods with thermal processes such as cooking or frying are not recommended. Their use has been banned or limited in some countries because of their harmful effects on human health.^136^ TBHQ (*tert*-butylhydroquinone) (E319), also very common, has applications in meats, margarines, cereals, and oils, but it is not efficient in the bread and pastry industry. Gallate compounds (E310–E312) are used alone or in combination with BHT and BHA. They have very limited, case-specific applications but can be used in dried milk, fats, oils, nut butters, potato products, chewing gums, cereals, meats, nuts, and food supplements. Other EFSA-approved chemical antioxidants include ascorbic acid and derivatives (E300–E304), EDTA (E385), erythorbic acid (E315), sodium erythorbate (E316), citrates (E330–E380), lactates (E325–E327), and tartrates (E334–E354).^134^

Ascorbic acid (E300) is a powerful antioxidant that can be used in practically used worldwide. It is commonly used in combination with artificial antioxidants such as BHT and BHA because of their excellent properties in regenerating other antioxidants. It also has a strong association with tocopherols.^19^ Sodium ascorbate (E301), used in several foods, was tested in fermented dry sausages to prevent oxidation of proteins and lipids. However, its application as a substitute for nitrites in foods is very limited due to its pro-oxidant behavior.^137^ Calcium ascorbate (E302) is used in dairy products, cured or cooked meat products, to dip freshly cut fruit and prevent browning, to intensify the color of cured meat, and to act as a synergist for other antioxidants.^138,139^ Fatty acid esters of ascorbic acid (E304) can be used in dairy products, chewing gums, cereals, meats, desserts, salads, sauces, and others.^140^ Erythorbates (E315–E316) are mostly dissipated in cured meats, frozen fruits, vegetables, oils, fats, seafood, and fish with the function of reducing the formation of nitrosamines during curing or cooking.^141^ Sodium lactate (E325) and potassium lactate (E326) are used as antimicrobials in processed and unprocessed meats, sausages, meat burgers, and fish.^142−144^

Citric acid (E330) is a very strong synergist, especially with ascorbic acid and chitosan. It also prevents browning of foods and increases their shelf life, since it also acts as a chelating agent.^145^ Another strong synergist with organic acids, specifically with citric, tartaric, malic, and lactic acids is sodium citrate (E331), which in turn has a chelating, pH buffering, retarding, antimicrobial, flavor-enhancing, and antioxidant effect, being mainly used in meats and skimmed milk.^146^ Potassium citrate (E332) and calcium citrate (E333) are used in jellies, marmalades, and cheeses as emulsifying agents, pH buffers, sequestrants, and antioxidants and are mainly used to modify intense flavors.^1^ Tartaric acid (E334) and its derivatives, sodium (E335), potassium (E336), and calcium (E336) salts, are used in chocolates, marmalades, gelatins, canned food, and fresh pasta as well as in cheeses, fats, oils, meats, and sausages.^1,147^ Phosphoric acid (E338) and its salts synergize with citric acid to prevent fat oxidation and are used in soft drinks, fruit jellies, cheese, and yeast powders.^148^ Sodium phosphate (E339) has chelating and antimicrobial effects, which are enhanced by synergy with nisin and is applied in pasta, meat, powdered milk, fruit, cheese, snacks, and ready-made desserts. Potassium phosphate (E340) is used in meats, breads, pasta, powdered juices, eggs, pasta, and sausages, calcium phosphate (E341) is used in the baking industry, fruit preserves, powdered juices flour, cheese, and porridge, and with less representation, ammonium phosphate (E342) and magnesium phosphate (E343), which can be used in breads, pasta, cookies, and pancakes. All are considered GRAS molecules.^1,19,38,141^ Adipic acid and its salts are used in cheeses, jellies, and canned fruit and is used synergistically with sodium metabisulfite (E223). Succinic acid is applied to chicken meat, dairy products, and cooked foods.^1,38,141,148,149^ Calcium disodium EDTA (E385) is used in foods such as poultry meats, processed meats, vegetables, fruits, beer, and fruit juices because it is a metal chelating compound, has antioxidant properties, and when acting with potassium sorbate, lysozyme, nisin, monolaurin, and monocapin increases its antimicrobial potential. It is also often added with ascorbic and citric acid, lecithins, BHA, BHT, and PG (propyl gallate) for the purpose of anointing fats but also in fresh and processed meats, fish, sauces, cereals, and seafood.^1,19,38,141,150^

In terms of natural antioxidant additives, rosemary extract (E392) is an example of the use of plant extracts by the food industry for food preservation, thus allowing the search for and possible use of other sources of natural antioxidants. Phenolic compounds, such as carnosic acid, rosmarinic acid, carnosol, and rosmaridiphenol, among others, that compose the rosemary extract and possess a great antioxidant power,^28^ as it allows a wide range of applications in the European Union, namely, in dehydrated milk, fats, processed potatoes, fine pastry, processed and heat-treated meat, fish, and eggs, mustard, soups, sauces, and food supplements, among others. It has not been assigned an admissible daily intake (AID) value, given its safety, and is considered a *quantum Satis* (it can be added to food in the quantity required to achieve the desired technological effect). It can also be used synergistically with other antioxidants such as nisin, polyphenols, BHA, and BHT to boost its antioxidant effect.^19,151−156^

#### 3.2.1 Natural Antioxidant Preservatives

The interest in natural antioxidants has grown exponentially, and their importance both *in vivo* and in food is undeniable. Natural and even functional additives that can be found in different plants, fruits, algae, and mushrooms have been presented as alternatives to synthetic additives in order to fortify and stabilize foods. Some compounds, such as vitamins, polyphenols, and carotenoids can even show similar protective effects to BHT and BHA, being described as among the most relevant natural antioxidant compounds.^19^

Polyphenols are known to have a powerful antioxidant capacity, with promising effects on human health, and due to these properties and the ability to preserve food as well as their good acceptance by consumers (as they are natural molecules), their use is attractive for the food industry, especially when it comes to replacing synthetic additives.^157^ The main groups of polyphenols are phenolic acids (hydroxybenzoic and cinnamic acids), flavonoids (anthocyanidins, flavones, flavonols, isoflavones, flavanols, and flavanones), lignans, and stilbenes.^98,99^ Among these phenolic compounds, some stand out more than others and can be added to foods individually, after purification of the molecules or used as plant extracts, taking advantage of the synergistic effects between them.^11^ Polyphenolic plant extracts, such as those from rosemary, previously mentioned and identified as a food additive have been used to act as antioxidants in foods. Despite the advantages of synergy between compounds, preference is often given to specific molecules by industries. In this sense, it is believed that carnosic acid, a derivative of the hydroxybenzoic acid constituent of rosemary extract, has the most significant antioxidant power, being used in oils, animal fats, sauces, roasts, meat, and fish burgers, among others.^158,159^ Ferulic acid, a hydroxycinnamic acid, is used as an antioxidant and precursor to other preservatives and has also been employed as part of food gels and edible films.^160,161^ Catechin (flavan-3-ol) can be added directly to food, combined with other natural substances as well as encapsulated, thus potentiating and amplifying its effects.^162^ Ascorbic acid (E300), i.e., vitamin C, can stabilize lipids and oils by helping to regenerate phenolic oxidants and tocopherols that have undergone oxidation. EFSA determined that its consumption is safe and did not define an ADI.^19,163^

Another group of natural food additives with antioxidant potential are carotenoids, although their use is limited because they are very susceptible to oxidation by light. The most common in food are lycopene, lutein, and β-carotene. They are used in foods such as meat, fish, fruit, cereal products, pastries, dairy products, and many others.^134^ The most widely used carotenoid in food is lycopene (E160d), found mainly in tomatoes, but it is not widely used as a food antioxidant. On the other hand, β-carotene (E160a) is used as an oxygen singlet in dairy products, bakery products, and eggs, among others.^149^ Astaxanthin is a carotenoid pigment not approved by the EU; this colorant has a high antioxidant capacity and is currently being studied as a possible food preservative. The use of carotenoids in the food industry must guarantee the stability of these molecules during storage.^164^ The union of carotenoids with ascorbic acid or vitamin E helps in synergies, and this mixture (either synthetically or extracted from plants and fruits) has been analyzed by the EFSA Scientific Panel, rejecting any toxicity from their consumption.^165^ Another compound with which ascorbic acid can act are tocopherols, acting as very strong antioxidants. They are used in films and coatings as well as in additives (E306–E309), exerting mainly an activity against lipid peroxidation and rancidity in oils, meats, vegetable oils, and dairy animal fats, among others.^149,166−170^

There are also other carotenoids that are used as food additives. Annatto, for example, is a mixture of several carotenoids extracted from the *Bixa orellana* shrub, with bixin being the most prominent one. Although annatto is used as a food colorant, it can also be used as an antioxidant, and its use against auto-oxidation of triacylglycerols in seeds has been reported. Other reports have suggested that synergistic links between tocopherols and norbixin allow for an increase in antioxidant potential, the latter being recognized as a stronger antioxidant than the former. Its use is permitted in the EU with an ADI of 0.065 mg/kg bw and is identified with the code E160b.^171^ Lutein has different biological functions, justifying its application in the cosmetic industry for its antioxidant capacity, especially for inhibiting lipid autoxidation in skin cells. Its activity is even stronger than that of β-carotene. Its use in the food industry according to EFSA is limited to being a coloring additive in baked goods, bread-based foods, soft drinks, and also as sauces and confectionery, not allowing its use as a food antioxidant.^19^

### 3.3 Antibrowning Agents

During the various stages of food production, several changes can occur in foods, such as browning processes. In this sense, additives that can counteract this reaction are important to maintain the stability and preservation of foods, and the most commonly used are sulfates. Going against the current focus, natural alternatives to these synthetic additives are compounds such as erythorbic acid (E315), used in beverages for flavor preservation; 4-hexylresorcinol (E586), an organic compound allowed only in shrimp, and an amino acid, l-cysteine (E920). Their effect is based on the reconversion of quinone intermediates to the phenolic form and reactions with quinolone intermediates to inhibit the formation of further compounds.^1,172^ Calcium lactate is also used to inhibit the browning of foods, especially fruits, by maintaining their structure. It is also applied synergistically with phosphates to improve their antioxidant capacity in meat.^173,174^

## 4 Potential Compounds with Dual Activity

**Figure 3:**
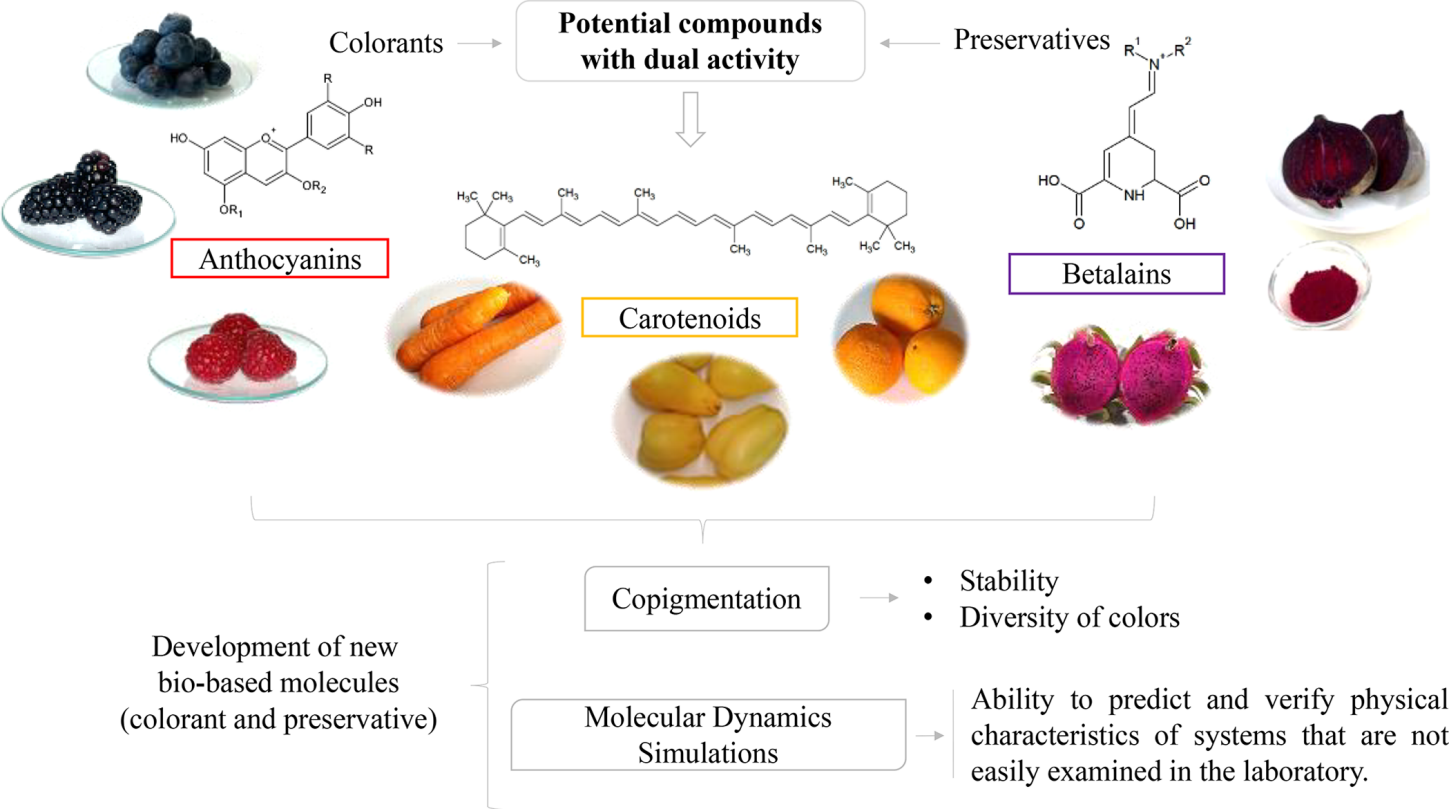
Schematic illustration of the development of new molecules, through new chemical approaches, with modification of already known natural molecules to develop a better and double performance (dye plus preservative).

The food industry is in search of technological advances as well as solutions, applications, and methodologies to obtain new products that respond to the needs and demands of consumers. The importance of preservative and color additives for the preservation and marketing of food products has led to the search for new solutions and/or improvement of existing alternatives. In nature, there are compounds that can have the capacity not only to stabilize or improve color but also to prolong the shelf life of the product. The evaluation of different natural matrixes that could have the ability to provide color and, at the same time, could act as preservatives has been of great interest in the food industry. Anthocyanins, carotenoids, betalains, and their derivatives are among the compounds that may provide these properties, owing to their pigmentation, antioxidant capacity, and/or antimicrobial activity (Figure 3).^14,175^ For instance, Ab Rashid et al.^176^ have recently developed an antibacterial food colorant based on anthocyanins from *Clitoria ternatea* flowers, showing a broad-spectrum of antibacterial activity and being able to maintain the color for 21 days at temperatures from −20 to 4 °C, making it an alternative as a biopreservative and colorant to be used in food products. Another study investigated the antimicrobial properties of eight food colorants extracted from plants (*Acacia catechu* L., *Bixa orellana* L., *Cassia auriculata* L., *Embillica officinalis* Gaertn, *Punica granatum* L., *Rubia tinctorum* L., *Tagetes erecta* L., and *Terminalia chebula* Retz.), concluding that the red pigments had better antibacterial activity, in contrast to the yellow pigments which showed better antifungal activity. This study also analyzed the antioxidant activity, where the red pigments showed again better activity than the yellow ones; these features would allow the incorporation of these dyes in food providing preservative properties.^177^ The phenolic composition of methanolic and ethanolic extracts of Murta (*Ugni molinae* Turcz) fruit was studied for evaluation of their antioxidant and antimicrobial activity; ethanolic extracts showed high antioxidant capacity, while methanol extracts yielded inhibitory activity against *Escherichia coli* and *S. typhi* bacteria similar to that of standard antibiotics, thus opening prospects to explore these extracts as potential biopreservatives with coloring capacity owing to their high anthocyanin content.^178^ In another study, the antibacterial and antifungal activity of a curcumin microcapsule was evaluated against some foodborne pathogens and spoilage microbes, including *Escherichia coli*, *Yersinia enterocolitica*, *Staphylococcus aureus*, *Bacillus subtilis*, *Bacillus cereus*, *Aspergillus niger*, *Penicllium notatum*, and *Saccharomyces cerevisiae*. The microcapsule showed a broad-spectrum inhibitory effect against all these microorganisms, especially against Gram-positive bacteria; furthermore, its antifungal activity was much better than the antibacterial activity. These results give insights into the possibility of using microencapsulated curcumin as a high potential colorant and preservative in the food industry.^179^

Betalains are also a good example of natural food colorants, exhibiting not only prominent coloring attributes but also a range of biological activities, such as antioxidant and antiradical ability, conferring protection against oxidative damage, antimicrobial properties, as well as antiproliferative, cytotoxic, and neuroprotective capacities, providing health benefits concomitantly with their coloring and preservative capacity.^180−184^

Another important group of compounds with dual activity are carotenoids. Some of the most important are α-carotene, β-carotene, and lycopene, which are already approved and used as food colorants. They not only provide color and/or food preservation, but their high antioxidant power makes them excellent natural food additives, with no toxicological threat in the amounts necessary for their use in food.^13^ β-carotene, a precursor of vitamin A, has been successfully added to various foods, especially functional beverages and nutraceuticals, and its application in new and existing products is expected to increase in the future. Lycopene, one of the main phytochemicals in tomatoes, has been used to increase stabilization, improve color, and provide health benefits when incorporated into minced meat.^134,185^

However, it is important to emphasize that to obtain functional benefits, such as antioxidant and/or antimicrobial activity, the concentrations of compounds commonly used as dyes are markedly different (usually higher) from those used for dye purposes, not meeting the requirement of acceptable daily intake (ADI) imposed by regulatory agencies so as not to pose health hazards.^5^ This confirms the need for research into new natural matrixes, which have coloring and preservative capacity, and may jointly provide beneficial properties to the consumer and a challenging opportunity for the scientific community to solve, through the development of new biobased molecules, the next generation of food additives, through new chemical approaches, modifying the natural molecules already known so that they develop a better and double performance (dye plus preservative).

Noncovalent complexation is a naturally occurring process and is the mechanism mostly responsible for stabilizing and enhancing blue, violet, and red colors (e.g., anthocyanins) in flowers, vegetables, and fruits as well as in food products derived from them (wines, jams, and syrups). The concept of copigmentation has been refined over the years, and the atomistic perception of it has been essential to complement it by elucidating color diversity in nature and its stabilization process in nature.^186^ The increased interest in copigmentation has been remarkable, particularly by the food industry, in order to improve the color palette. In view of its mastery and use through the addition of copigments to food products, accurate (computer-aided) control of supramolecular assemblies of noncovalent supramolecular copigments is essential.^187^ In this sense, the copigmentation with antioxidant/antimicrobial molecules can also be explored, and the use of new tools and cheminformatic models can support the development of unique hybrid compounds with dual function (staining and preservation), based on the knowledge of numerous biomolecules spawning new biobased molecules as the next generation of food additives.^188,189^

## 5 Molecular Dynamics Simulations: Challenges and Opportunities

The delivery of technology and biofunctionality in all-natural processed foods is an area of increasing fundamental and technological interest.^189^ Physical properties and stability are critical for delivering safe and healthy food to consumers, and therefore it is a topic that has attracted food scientists for a long time.^190^ Food is a complex system that undergoes many physical and chemical transformations during processing and subsequent storage. These transformations are governed by molecular motions within the final product and reflect its overall stability and perceived quality.^191,192^

Simulation-based research has been going on for a few decades, and computer simulations of biomolecular systems have been growing rapidly in recent years. Characterized by detailed information about the atomic/molecular structure and not subject to physical fallacies, molecular dynamics (MD) simulation has been touted as the most promising tool for the characterization of multicomponent systems and has proven to be a useful methodology for investigating complex geometries and molecules as well as studying structural and dynamical properties.^193^ MD simulation has enabled the study of many biological systems over the past decade, from small molecules, such as anesthetics or small peptides, to very large protein complexes, such as the ribosome or virus capsids.^194−197^

Due to the complexity of biological and nonbiological systems, MD simulations have come under great interest for their ability to predict and verify experimental results, providing an opportunity to study the physical characteristics of systems that are not easily examined in the laboratory. Research aimed at improving MD algorithms so that they can simulate the folding and unfolding of proteins is one example of the study of these features.^198,199^ In addition to biological applications, MD simulations have been used to study the physical characteristics of nonbiological nanoparticles.^200^ Comparisons of simulation and experimental data serve to test the accuracy of the calculated results and provide criteria for improving the methodology.^201^ Another significant aspect of the simulations is that although the potentials used in the simulations are approximate, they are entirely under the control of the user, so that by removing or changing specific contributions, their role in determining a particular property can be examined. Intensive computational processing power is required to simulate a complex system using MD simulations. With the available tools, it is only possible to simulate a dynamic system in a time interval of the order of femtoseconds. However, it is expected that, with the development of better MD simulation software and significantly faster computer hardware, MD simulation will become faster and more accurate and will be performed for more extended periods of simulation time.

Given the current demands for cleaner food, the preference for natural additives, and the toxicity and safety issues with synthetic additives, research into natural compounds that fulfill dual functions is emerging. However, such compounds may take time to become part of the additives allowed for use by regulators, noting that in addition to research for these new molecules, they must undergo thorough toxicity and safety evaluation before their use becomes permissible for consumption. A promising alternative would be to exploit already existing natural additive biomolecules with accepted worldwide use and, through new chemical approaches and modification of these molecules, develop unique hybrid compounds with dual function (coloring and preservation). This is because usually their individual use is directed only to one function, and to exert other activities (antioxidant and/or antimicrobial) the concentrations are usually higher than those used for dyeing purposes, which may pose health hazards.^5^ In this sense, and despite some limitations of molecular dynamics simulations, their exploitation for research and development of these hybrid molecules, predicting and verifying the experimental results, allow one to study certain physical characteristics that are not easily examined in the laboratory and very promising and can help and accelerate research and studies on a topic that is currently fundamental.

## 6 Closing Remarks

The controversy and ambiguity among chemical additives have encouraged the search for natural alternatives, which are easily accepted by consumers. Combining the trends of health-oriented eating and lifestyle, exercise, safe, unprocessed, and healthy eating, there is a growing tendency to reduce the addition of additives in food or to replace them with novel natural additives. Nevertheless, the role they play in maintaining and/or enhancing food quality standards still justifies their application in the distinct production and storage processes and, therefore, the search for innovative and effective solutions.

Although natural compounds tend to be safer than those produced synthetically, studies concerning toxicity, carcinogenicity, and others must be conducted to ensure their safety. Another major limitation is their actual effectiveness is that if a large amount is required for their ability to be exerted, it may lead to changes in the food in terms of appearance, taste, and/or texture, so it is important to determine their effective outcome properly, remembering that if larger quantities of natural additives are required than synthetic ones, their use may not be cost-effective or recommended. In addition, the lack of appropriate legislation for natural additives (regulated identically to synthetics) is a barrier to the approval of new compounds/extracts and cuts across both, making it difficult to bring new compounds to the market. Their delay is also justified by all the trials that must be conducted to ensure safety standards and achieve a proper ADI. Synergistic effects and multiple functions simultaneously are common in natural additives; however, some may be incompatible with others, natural or artificial, as well as with the constituents of food itself, so that their use may be impeded. Despite the described limitations, additives of natural origin are believed to be the future of food preservation allowing shelf life extension and preventing food loss. By overcoming the limitations, better, safer, and more efficient natural additives will be achieved and/or new artificial compounds can be discovered as well as synergistic effects between the currently approved additives. It should be noted that all these advances and hypothetical novel food additives are regulated by the governing bodies, and the possible demystification, increased efficiency, and ease of access to other types of food preservation methods may also limit the need for additives. Concomitantly, we can count on the advance of computer and computational methodologies, which increasingly serve as tools to support experimental methodologies, through *in silico* assays. These complement the experimental assays that, besides making research less painful and time-consuming, provide important data that would otherwise be difficult to obtain. Other strategies to food preservation that may involve a lighter additive load are dual-function additives, resulting from the combination of two types of additives to perform distinct functions in foods, such as preservative colorants. Such compounds would have undoubtedly to be safe, inexpensive, and not alter food. Although difficult to accomplish, we have been much further away from finding them.
